# A Simple and Reliable Health Monitoring System For Shoulder Health: Proposal

**DOI:** 10.2196/resprot.2584

**Published:** 2014-02-26

**Authors:** Shuo-Fang Liu, Yann-Long Lee

**Affiliations:** ^1^National Cheng Kung UniversityDepartment of Industrial DesignTainan CityTaiwan; ^2^Ling Tung UniversityDepartment of Technological Product DesignTaichung CityTaiwan

**Keywords:** health care service, network platform, shoulder health, scale design, PHP

## Abstract

**Background:**

The current health care system is complex and inefficient. A simple and reliable health monitoring system that can help patients perform medical self-diagnosis is seldom readily available. Because the medical system is vast and complex, it has hampered or delayed patients in seeking medical advice or treatment in a timely manner, which may potentially affect the patient’s chances of recovery, especially those with severe sicknesses such as cancer, and heart disease.

**Objective:**

The purpose of this paper is to propose a methodology in designing a simple, low cost, Internet-based health-screening platform.

**Methods:**

This health-screening platform will enable patients to perform medical self-diagnosis over the Internet. Historical data has shown the importance of early detection to ensure patients receive proper treatment and speedy recovery.

**Results:**

The platform is designed with special emphasis on the user interface. Standard Web-based user-interface design is adopted so the user feels ease to operate in a familiar Web environment. In addition, graphics such as charts and graphs are used generously to help users visualize and understand the result of the diagnostic. The system is developed using hypertext preprocessor (PHP) programming language. One important feature of this system platform is that it is built to be a stand-alone platform, which tends to have better user privacy security. The prototype system platform was developed by the National Cheng Kung University Ergonomic and Design Laboratory.

**Conclusions:**

The completed prototype of this system platform was submitted to the Taiwan Medical Institute for evaluation. The evaluation of 120 participants showed that this platform system is a highly effective tool in health-screening applications, and has great potential for improving the medical care quality for the general public.

## Introduction

Around the world, some parts of the population live in areas distant from primary medical care facilities. Generally, these people have limited access to receiving proper preventive medical care, which often results in delayed treatment [1-4].

With the rapid advancement of telecommunication technology, some potential applications to certain medical practices, previously deemed infeasible and impractical, have recently been opened up. Further, this also allows an important channel for residents in rural areas to seek medical or diagnostic advice. For instance, with the advent of telecommunication technology, physicians are able to perform medical diagnostics, and enable medical devices (MRI, CAT scans, etc), using technologies such as video conferencing and the Internet, even though the patient is hundreds or thousands of miles away [5].

Internet-based medical technology has quickly become a critical part of modern health care systems and medicine [6-8]. Some good examples are in the fields of radiology, cardiology, dermatology, and family health [9-11].

The feasibility of using Web-based applications to perform medical diagnostics is limited [12,13]. This is due to the restricted capability and reliability of the program itself, and also the complexity and the nature of no 2 patients being identical. However, it is undeniable that Web-based medical applications can help a patient who lives in a remote location and has limited access to medical care facilities. In addition, it may also serve as a tool to alert a patient to seek care without delay.

The aim of this paper is to propose a simple, easy to implement, efficient, and reliable system for a telemedicine service as a preliminary medical diagnostic tool. The system is designed to enable the user to record the results of the diagnostic, and the results could be used by a physician in conjunction with future diagnostics. This is an application purposely built with a user-friendly, graphical interface and various services are implemented as dynamic Web pages. In addition, the application is developed with specific emphasis on patient privacy and ease of use. It is vital to create an environment in which the patient does not feel intimidated [14,15]. The system design considerations, the description of the system, and the results of the system validations are presented in the following sections.

## Methods

### System Design

The initial step was to conduct a comprehensive analysis of the study’s scenario. Two types of users were identified to use the system: physicians in medical centers, which hosted the system platform, and patients located in remote and underserved areas that have concern for his/her health. Both users were required to have access to any active Internet connection and the ability to run popular Web browsers such as Internet Explorer and Mozilla Firefox. Since these Web browsers are supported by multiple operating systems, the users were able to access the application from any computer system with ease. In addition, the system design architecture was not limited to one unique type of medical symptom. The system design architecture was applicable to all types of medical symptoms or conditions with small updates to certain parts of the system. In this study, the medical symptoms due to neck and shoulder disorders were used as an example, and a proven Shoulder Fatigue Scale-30 Items (SFS-30) diagnostic scale [15] was used to determine the severity of patient neck and shoulder discomfort. In addition, we investigated how the SFS-30 scale was to be improved and applied, so this was not an experimental study, and it does not belong to randomized controlled trials.

### System Management

We provide a hypothetical situation based on a patient who is suspected of suffering some form of neck and shoulder disorder, and is not sure the symptoms are severe enough to see a physician. This patient chose to get an initial diagnosis using a Web-based application from the medical center s/he preferred. The patient may have needed to pay a small fee to access the Web-based diagnostic application, and was required to input all the symptoms and a description of his/her concerns. Next, the data was submitted to the server for analysis and reviewed by the medical center’s attending physician. The final diagnosis was then provided to the patient in electronic form. The patient accessed the Web-based system to see the diagnosis and determined if s/he was required to seek treatment.

Note that the system service platform also provided a link that the patient was able to use to communicate with the medical center’s physicians. All Web-based medical diagnostic systems have certain limitations. Hence, when the final diagnosis was prepared, either comprehensive or broad, the patient was able to contact the medical center’s physician swiftly to get additional information, clarification, and prevent further deterioration of the patient’s medical-related conditions.

### Interface Design

The system interface was designed with the assistance of 10 experts in the area of interface design, information system design, and prototype testing. The system’s overall performance and satisfaction were acquired through questionnaires (questionnaires are available upon request). Lastly, the Heuristic Evaluation Method was used to determine the best method in diagnosing neck and shoulder pain symptoms, and also used to further refine and improve the overall system design.

## Results

### System Architecture

**Figure 1:**
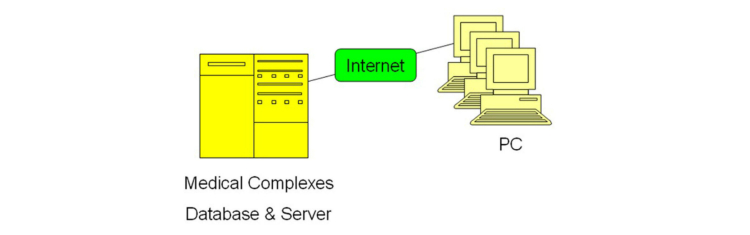
General architecture of the system.

Figure 1 represents the different elements that make up the system architecture. The host center was located at the medical center’s local server. This server was a database for the system platform, and designed to be accessed simultaneously by multiple personal computers. The system was accessible by all medical staff members and current registered patients of the medical center.

### System Service

**Figure 2:**
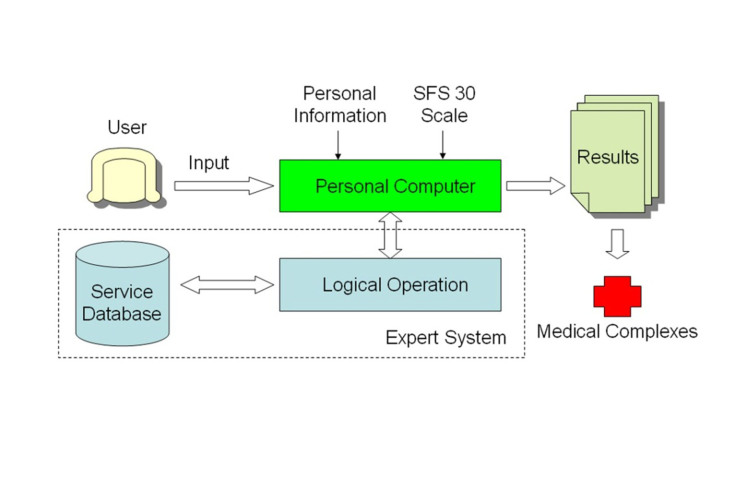
System services.

The system performed two primary functions. The first function was to evaluate the data provided by the user. Next, the analyzed data was used to develop the final diagnosis and medical advice. In order to perform these functions, the three major components required for this system to perform its task were (1) data (inputted by the user), (2) database (storage and retrieval of data), and (3) personal computer and internet network (to complete the expert system; Figure 2).

Table 1, shows the comparison of three common types of testing platform design development. Simple platform design is selected for its low development cost, no geographical location limitation, and ability to output the final result in PDF format.

For this research, the system platform was developed using the Microsoft Windows XP operating system, and is designed for the Internet Explorer 6.0 or newer browser. The Web page is best viewed with 1024 × 768 screen resolution. The Pietty software package version 0.3.27 was used to develop the system platform. This software package was selected for its simple user interface, customizable window display, its support of multiple languages, and its advantage of direct drag and drop file upload.

The diagnosis data is reformatted using a PHP class that allows PDF files to be generated with pure PHP, also known as FPDF, and saved in a commonly used PDF format. This enables the user to store the file electronically and readily accessible. In addition, this reduces paper usage, which is beneficial for the environment. Next, a commonly used Java script was added to detect an incomplete input field. This was done to ensure all required input fields had been entered properly. To ensure the system platform could be displayed and function properly, additional PHP code was added to ensure compatibility with all types of browsers (Table 2).

**Table 1:** Comparison of functional requirements in three types of testing platforms.

Functional requirements/types	Expert	Standard	Simple
Usage requirement	Physician	Onsite self-diagnostic	Web-based self-diagnostic
Target user	District hospital	Clinic	Public
Operating environment	Reside in user terminal	Reside in user terminal	Access through Internet
Programing language	JAVA	VB	PHP
Type of storage	None, printable	None, printable	PDF
Output format	Text, chart	Text, chart	Text, chart, figure
Development cost	High	Low	Low

**Table 2:** System development requirements of the testing platform.

Operating system	Ubuntu 4.1
Operating environment	Microsoft Windows XP Pro SP2
Browser	IE 6.0 and Newer
Development Tool	Pietty 0.3.27
Programming language	PHP/5.2.6-3, Apachie/2.2.11
Interface design	CSS
Chart design	Google Chart Tools, Flash
Data storage	MYSQL, FPDF
Testing	JavaScript
Analysis	SFS-30 questionnaire

### System Access Management

Since this is a Web-based application, all data was transmitted through the Internet, and hence there was a potential risk of the data being intercepted or manipulated by someone other than the user. To ensure the safety and confidentiality of the database, all users were required to register and obtain permissions from the system administrator prior to accessing the system platform. For all registered users, s/he had the confidence of accessing the medical information easily and safely.

The system administrator was also responsible for establishing the database with relevant medical information and inputting from physicians. This was essential for the user to obtain complete, up-to-date, and accurate information.

### Graphical User Interface

**Figure 3:**
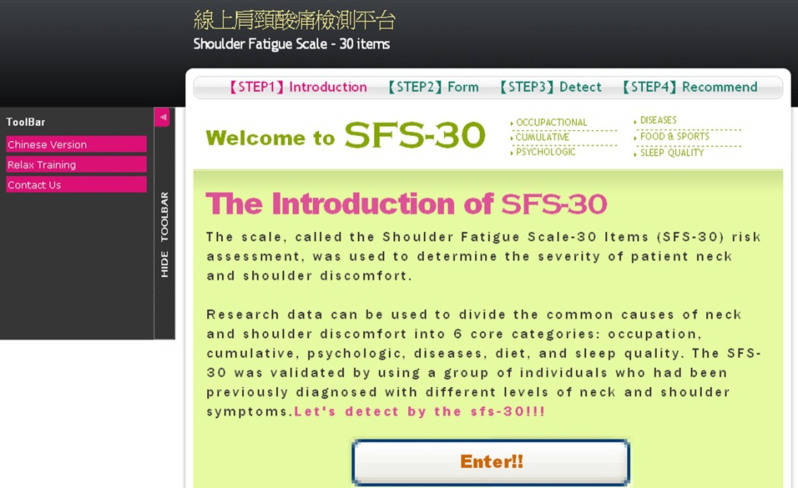
Main window.

The user interface is a user-center graphical interface design. The first thing the user saw after launching the SFS-30 website is shown in Figure 3. From the main window, the steps are clearly labeled and progress is shown at the top edge of the window frame. At the left side of the window, under the tool bar there are additional buttons, which enable the user to switch Web content in languages between English and Chinese, and also medical center contact information.

**Figure 4:**
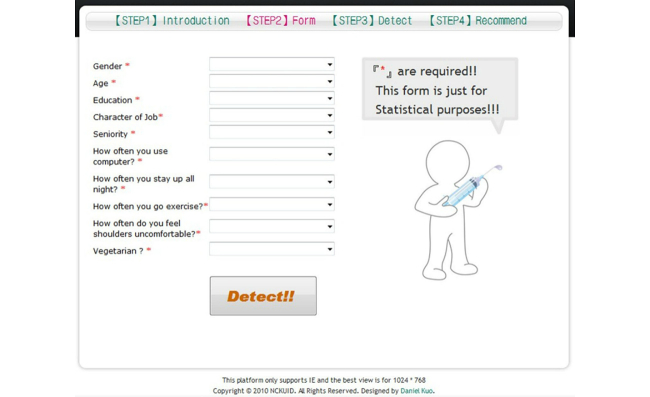
Personal information.

In Figure 4, Step 2, the user clicked on the “Enter” button shown in “Step 1, Introduction” and proceeded to the user’s information input section.

**Figure 5:**
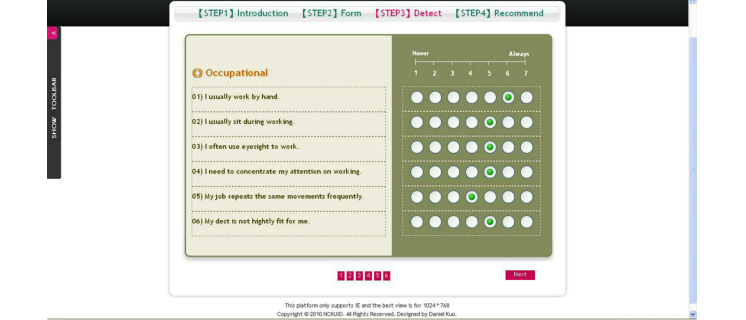
SFS-30 Scale.

Step 3 of Figure 5 is the input section required for the SFS-30 scale. Questions were asked regarding the user’s current body condition from level 1 to 7. The user was required to select/click the appropriate circle that best fits the user’s current physical condition.

**Figure 6:**
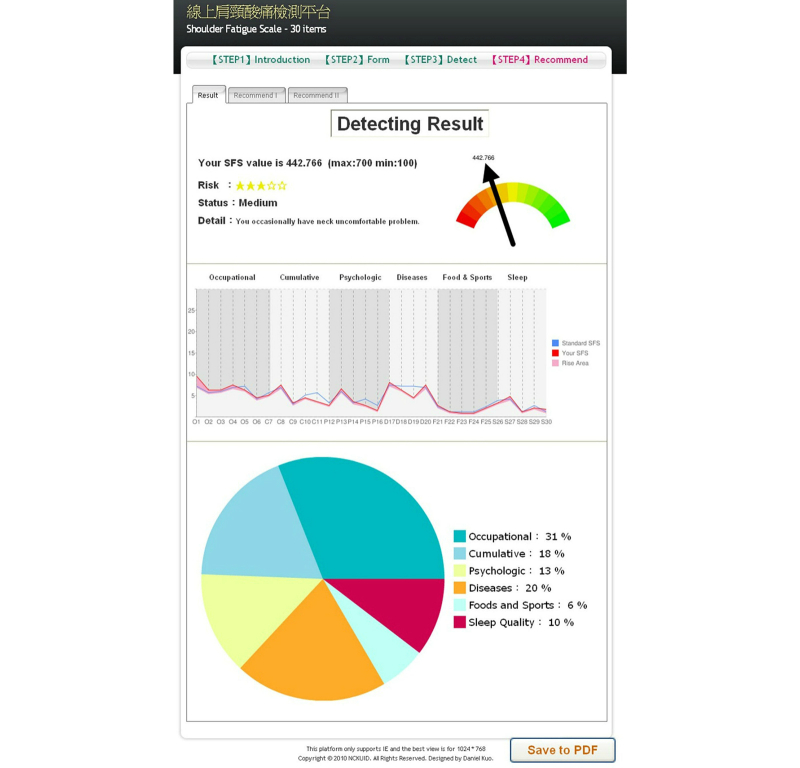
Results.

Step 4 of Figure 6 presents the findings and recommendations. The results were presented in multiple layers to help the user understand his/her medical conditions with ease, and provided medical recommendations. All the information was presented in such a way that the user could make a decision for the next course of appropriate action to treat his/her medical conditions.

### Validation and User Evaluation

When predicting or explaining the behavior of individuals, the “Intention model” is considered to be a complete model. The Intention model factors in attitude, beliefs, and affection, therefore predicting an individuals’ behavior. If someone wants to predict and explain whether a person will act in a specific manner, we have to understand their intentions.

Fishbein and Ajzen [16] proposed “Theory of Reasoned Action (TRA)”, which considers the antecedents of behavior is the Intention model, and impacts either or both of the “attitudes toward the behavior” and “subjective norms concerning the behavior”.

Based on the TRA and with the application of information system, Davis et al [17] proposed a model called “Technology Acceptance Model (TAM)”. This model contains two basic assumptions. The first is “perceived usefulness” (PU), where people think that using a particular system will be able to enhance performance. When the user perception level of usefulness of the system is higher, they will perceive the system more positively. The second is “perceived ease of use” (PEOU), in which people think that learning to use a particular system is easy. When users think learning the system is easy, they will also perceive the system more positively.

User evaluation and validation was conducted to evaluate the clinical trial of this system. The evaluation method used was a prototype system developed by Cheng Kung University Laboratory of Human Factors Engineering. The prototype system combined task-technology fit (TTF) and TAM models. Yen et al [18] combined and simplified the theory models of Goodhue and Thompson [19] and Dishaw and Strong [20]. Through the use of TAM and TTF to analyze the impact of the determinants of the subjects on the detection system, the system developed a more complete assessment. This prototype system was particularly suitable for this application. The validation was done using Structural Equation Modeling (SEM; LISREL version 8.51) to find the goodness of fit indices.

The evaluation and validation were conducted at Taipei Veterans General Hospital (Taiwan) in November 2010. There was a total of 120 patients who participated in this study. Among the 120 patients, there were 79 males and 41 females with an average age of 34-years old. Table 3 lists the demographic details of the patients.

The causal structure of the proposed research model was conducted using SEM. SEM is a modeling method that can handle a series or group of independent variables and the relationship between the dependent variable. In this study, LISREL 8.51 software was used to calculate the SEM fit indices. The recommended value and the numerical results of this study are listed in Table 4. From the table, most fit indices were found within the acceptable range. For instance, χ2/df =1.41, goodness-of-fit index (GFI)=0.92, adjusted goodness-of-fit index (AGFI)=0.89, root mean square error of approximation (RMSEA)=0.026, and (expectation for a) constant factor index (CFI)=0.96 are consistent with expected values.

**Table 3:** Demographic information of the respondents.

Demographic information		n (n=120)	Percentage
**Gender**			
	Male	79	65.8
	Female	41	34.2
**Age**			
	Under 20	5	4.2
	21-30	53	44.2
	31-40	22	18.3
	41-50	24	20
	51-60	13	10.8
	Above 60	3	2.5
**Education Level**			
	High school	5	4.2
	College	74	61.7
	Graduate school	39	32.5
	Doctorate/PhD	2	1.7
**Profession**			
	Typical white collar worker	51	42.5
	Athlete	8	6.7
	Service	31	25.8
	Housewife	13	10.8
	Porter	3	2.5
	Other	14	11.7
**Computer usage**			
	Do not use the computer	0	0
	Less than 1 hour/day	13	10.8
	1-4 hours/day	32	26.7
	5-8 hours/day	55	45.8
	More than 8 hours/day	20	16.7

**Table 4:** Measures of model fit for measurement model.

Measures of model fit	Recommended value	Recommended by	Research value
χ2	--	--	273.9
*df*	--	--	194
χ^2^ / *df* ^a^	<3	Hayduk	1.41
GFI^b^	>0.9	Scott	0.92
AGFI^c^	>0.8	Scott	0.89
CFI^d^	>0.9	Bagozzi & Yi	0.96
RMSEA^e^	<0.05	Bagozzi & Yi	0.026

^a^Chi-square value divided by the degrees of freedom

^b^Goodness-of-fit Index

^c^Adjusted Goodness-of-fit Index

^d^(Expectation for a) constant scale factor index

^e^Root mean square error of approximation

From the reliability analysis, the overall scale of the Cronbach α value was found to be 0.946 (n=120). This indicated that the result of this questionnaire (questionnaires available upon request) had good internal consistency.

Next, composite reliability (CR) was conducted to check the consistency of internal dimension. The higher the CR value, the higher the correlation between the observed variables. Hair et al [21] pointed out the confirmatory factor analysis of SEM, the reliability of each dimension must be greater than 0.7. From Table 4, all six dimensional CR values were found greater than 0.7. These CR values indicated that the dimensions of this questionnaire were in good internal consistency.

Average variance extracted (AVE) is a measure of the shared or common variance in a latent variable (LV), and the amount of variance that is captured by the LV in relation to the amount due to its measurement error. In another words, AVE is a measure of the error-free variance of a set of items. Per Fornell and Larcker [22] the recommended AVE value should be greater than 0.5. From Table 5, the AVE values for all six dimensions were found to be greater than the recommended value of 0.5. This showed that the questionnaire has a certain convergent validity.

Discriminant validity was used to calculate the degree of difference in dimensions and trait. Hair et al [21] indicated that if the square root of mean variance is greater than the correlation matrix, the various dimensions have good discriminant validity. Table 6 shows the coorelation coefficient matrix of LV.

**Figure 7:**
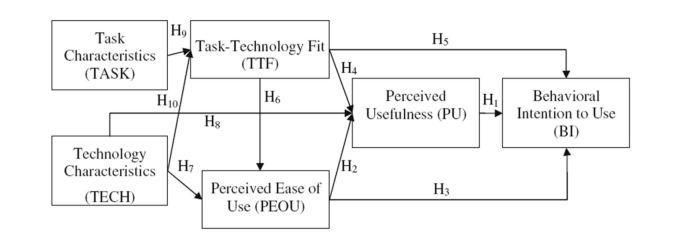
Integrated theoretical model of TTF and TAM [15].

The last stage of the validation process was to use LISREL to calculate the γ and β values. These values are used to explain the observed variables and LV, and relations between each LV. Table 7 summarizes the hypothesis, and Figure 7 demonstrates the results of the verified hypothesis.

Textbox 1 shows the results of the verification of the following hypotheses that are supported.

**Supported hypothesis results.**

H1: User PU and BI show positive correlation.

H2: User PEOU and PU show positive correlation.

H3: User PEOU and BI show positive correlation.

H7: The TECH and user PEOU show positive correlation.

H8: The TECH and user PU show positive correlation.

H9: The TASK of improving the user’s neck and shoulder pain symptoms and the TTF show positive correlation.

H10: The TECH of improving the user’s neck and shoulder pain symptoms and the TTF show positive correlation.

**Table 5:** Reliability analysis.

Latent variable		Observed variable	Factor loading	Measurement error	Composite reliability	Average variance extracted
**Behavioral intention (BI)**						
		BI1	0.86	0.24	0.872	0.773
		BI2	0.83	0.18		
**Perceived usefulness (PU)**						
		PU1	0.61	0.03	0.939	0.857
		PU2	0.57	0.15		
		PU3	0.67	0.07		
		PU4	0.72	0.18		
**Perceived ease of usefulness (PEOU)**						
		PEOU1	0.84	0.22	0.924	0.781
		PEOU2	0.79	0.17		
		PEOU3	0.9	0.29		
		PEOU4	0.86	0.27		
**Task-technology fit (TTF)**						
		TTF1	0.75	0.25	0.898	0.754
		TTF2	0.63	0.26		
		TTF3	0.72	0.19		
		TTF4	0.78	0.24		
**Technology characteristics (TECH)**						
		TECH1	0.65	0.16	0.927	0.840
		TECH2	0.52	0.12		
		TECH3	0.68	0.11		
		TECH4	0.56	0.07		
**Task characteristics (TASK)**						
		TASK1	0.87	0.24	0.879	0.646
		TASK2	0.79	0.27		
		TASK3	0.84	0.28		
		TASK4	0.85	0.75		

**Table 6:** The correlation coefficient matrix of the latent variable.

	BI	PU	PEOU	TTF	TASK	TECH
BI^a^	0.88					
PU^b^	0.73	0.93				
PEOU^c^	0.69	0.74	0.88			
TTF^d^	0.58	0.66	0.72	0.87		
TASK^e^	0.37	0.62	0.52	0.57	0.80	
TECH^f^	0.77	0.76	0.76	0.71	0.51	0.92

^a^Behavioral intention

^b^Perceived usefulness

^c^Perceived ease of usefulness

^d^Task-technology fit

^e^Task characteristics

^f^Technology characteristics

**Table 7:** Structural model results.

Hypothesis	Hypothesis (H)	β	t Statistic	Results of hypothesis testing
PU^a^→BI^b^	H1	0.52	7.46	Supported
PEOU^c^→PU	H2	0.26	3.52	Supported
PEOU→BI	H3	0.21	3.16	Supported
TTF^d^→PU	H4	0.12	1.32	Not supported
TTF→BI	H5	0.19	2.92	Not supported
TTF→PEOU	H6	0.13	1.30	Not supported
TECH^e^→PEOU	H7	0.47	4.28	Supported
TECH→PU	H8	0.35	3.33	Supported
TASK^f^→TTF	H9	0.22	3.30	Supported
TECH→TTF	H10	0.64	8.65	Supported

^a^Perceived usefulness

^b^Behavioral intention

^c^Perceived ease of usefulness

^d^Task-technology fit

^e^Technology characteristics

^f^Task characteristics

## Discussion

### Self-Diagnostic Tool

Historically, a simple medical self-diagnostic scale tends to have low accuracy and minimal reference value. This type of self-diagnostic scale is also not readily available and printed on paper. At the present time, patients have limited options in receiving a proper medical diagnosis without seeing the physician in person. Hence, the Web-based, self-diagnosis system developed in this study will be a great alternative available to the patient.

The main finding of this study was achieved in developing a self-diagnosis system architecture that is beneficial to the general public. The system was developed with the primary objective in providing a low cost, self-diagnostic tool and can be accessed through any personal computer that is connected to the Internet. Further, the tool is developed with a user-friendly interface that is simple and intuitive to use without special instructions. Another important feature of the tool is that it enables users to save a copy of the diagnosis for their personal records, and can be used as a reference for future visits with the physician.

**Figure 8:**
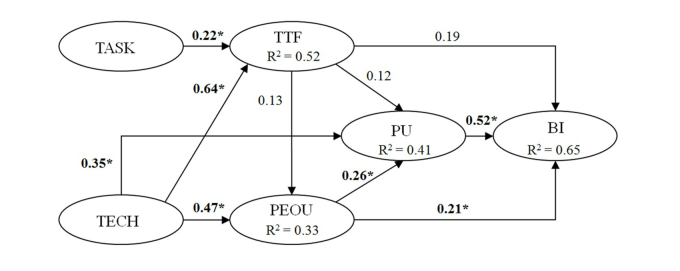
Structural model results (*correlation is significant at the 0.01 level).

The proposed system was tested using a hypothesis model developed by Yen et al [15]. The hypothesis model was based on TAM and TTF (Figure 8). This method was used to determine the factors that influenced the user’s decision to use the detection system. According to the result of the hypothesis testing, PU and PEOU had positive relations with BI. This shows that under the circumstances the detection system was not only easy to use, but will also boast the user’s desire to continue to use the detection system. Next, TECH significantly predicted the subject’s PU and PEOU. Thus, hypothesis H7 and H8 are supported. This result was not surprising due to the fact that previous studies, such as TAM, had shown that there is a significant relationship between PU and PEOU. Next, H8 indicated that the TECH is one of the external variables that significantly predict PU. This result contributes to previous research in which the effect of technology on PEOU is emphasized, but the relation between technology and PU is unnoticed [15].

On the other hand, hypotheses H4, H5, and H6 are not supported. Per TTF model definition, this is used to measure the degree to which a technology can assist an individual in carrying out his/her tasks. The findings suggested that users themselves may already know the causes of his/her neck and shoulder pain symptoms, and this is probably due to bad habits, or an undesirable working environment that he/she has limited or no control over it. Therefore, the user does not feel compelled to use the diagnostic system in treating his/her problems, even with knowing the system is simple and beneficial.

### Study Limitation

The accuracy of any diagnostic test depends on the inputs. Often, the user might not select the correct response and this could result in an inaccurate diagnosis. However, Liu’s [15] research indicated that an assessment scale could serve as a health-screening tool. Hence, even if the diagnosis may not be accurate; it can still provide some sort of warning message and the state of severity of the user’s current medical conditions. The physician can use the warning message from the diagnosis for a reference as well.

### Conclusions

The purpose of this study was to develop a medical, self-diagnostic system for neck and back pain patients. This system is supported by the inspection database, expert systems, and a decision-support mechanism. Upon the completion of the diagnostic, the system will generate a report consisting of charts, level of severity, and recommendations in a PDF format. Note that this report contains information based on research from medical doctors; hence, the information can be used to assist patient’s attending physician in developing the proper treatments.

In addition, the diagnosis identifies the potential root cause of the user’s neck and back pain symptoms, and provides recommendations that the user can choose to pursue in alleviating his/her conditions perhaps due to environmental factors or poor personal habits. In doing so, the diagnosis can aid the user in preventing his/her neck and back pain symptoms from becoming a health risk.

## Abbreviations

AGFI: adjusted goodness-of-fit index
AVE: average variance extracted
BI: behavioral intention
CR: composite reliability
CFI: (expect for a) constant scale factor Index
FPDF: a PHP class that allows PDF files to be generated with pure PHP
GFI: goodness-of-fit index
LV: latent variable
PEOU: perceived ease of usefulness
PHP: hypertext preprocessor
PU: perceived usefulness
RMSEA: root mean square error of approximation
SEM: structural equation modeling
SFS-30: Shoulder Fatigue Scale-30 Item
TASK: task characteristics
TAM: Technology Acceptance Model
TECH: technology characteristics TRA: Theory of Reasoned Action TTF: task-technology fit
TRA: Theory of Reasoned Action
TTF: task-technology fit
